# Comparison of Outcomes between Individuals with Pure and Mixed Lupus Nephritis: A Retrospective Study

**DOI:** 10.1371/journal.pone.0157485

**Published:** 2016-06-15

**Authors:** Titilayo O. Ilori, Nosayaba Enofe, Anju Oommen, Jason Cobb, Jose Navarrete, Demilade A. Adedinsewo, Oluwatobiloba Oshikoya, Helene Fevrier, Alton B. Farris, Laura Plantinga, Akinlolu O. Ojo

**Affiliations:** 1 Division of Nephrology, Department of Medicine, Emory University School of Medicine, Atlanta, Georgia, United States of America; 2 Department of Medicine, Morehouse School of Medicine, Atlanta, Georgia, United States of America; 3 Institute for Cardiovascular and Metabolic Disease, University of North Texas Health Science Center, Fort Worth, Texas, United States of America; 4 Hubert Department of Global Health, Rollins School of Public Health, Emory University, Atlanta, Georgia, United States of America; 5 Department of Pathology and Laboratory Medicine, Emory University School of Medicine, Atlanta, Georgia, United States of America; 6 Emory Transplant Center, Department of Surgery, Emory University, Atlanta, Georgia, United States of America; 7 Division of Nephrology, University of Michigan, Ann Arbor, Michigan, United States of America; Renal Division, Peking University First Hospital, CHINA

## Abstract

**Introduction:**

Lupus nephritis (LN) is a serious organ manifestation of systemic lupus erythematosus. Histologic overlap is relatively common in the six pathologic classes (I to VI) of LN. For example, mixed proliferative LN (MPLN) often includes features of classes III & V or classes IV & V combined. We performed a comparative evaluation of renal outcomes in patients with MPLN to patients with pure proliferative LN (PPLN) against pre-specified renal outcomes, and we also identified predictor of clinical outcomes among those with PPLN and MPLN.

**Hypothesis:**

Individuals with MPLN will have worse short-term renal outcomes compared to those with PPLN.

**Methods:**

We retrospectively reviewed 278 adult LN patients (≥18 years old) identified from an Emory University Hospital registry of native renal biopsies performed between January 2000 and December 2011. The final analytic sample consisted of individuals with a diagnosis of PPLN (n = 60) and MPLN (n = 96). We analyzed differences in clinical and laboratory characteristics at baseline. We also assessed associations between LN category and renal outcomes (complete remission and time to ESRD) with logistic and Cox proportional hazards models within two years of baseline.

**Results:**

The study population was predominantly female (83.97%) and African American (71.8%) with a mean age of 33.4 years at baseline. Over a median follow up of 1.02 years, we did not find any statistically significant associations between MPLN and the development of ESRD or remission when compared to patients with PPLN (adjusted HR = 0.30, 95% CI = 0.07, 1.26).

**Conclusion:**

There was no association between mixed or pure histopathologic features of LN at presentation and rate of complete or partial remission but higher baseline eGFR was associated with a lower probability of complete remission among patients with lupus nephriti**s.**

## Introduction

Lupus nephritis (LN) is one of the most devastating manifestations of systemic lupus erythematous (SLE). Most epidemiological studies report the incidence of LN among individuals with SLE around 35%, with a lifetime risk of up to 60%. This high incidence accounts for a significant number of individuals progressing to end stage renal disease (ESRD). [1, 2] Plantinga et al estimated the incidence rate of ESRD among newly diagnosed SLE patients in Georgia to be 11.1 per 1000 patient-years.[3]

The International Society of Nephrology and Renal Pathological Society ISN/RPS revised the WHO classification of LN in 2003, dividing LN into class I to VI.[4] Proliferative LN is defined as either Class III or Class IV. Proliferative LN can also co-exist with membranous LN (class V). According to the new ISN/RPS classification system, when a diffuse membranous LN occurs with an active lesion of class III or class IV, both diagnoses are to be reported, thus creating what we have termed “mixed proliferative lupus nephritis (MPLN)”.[4] MPLN has been reported in 22–31% of cases with LN. [5, 6] Together these classes III, IV, alone or in combination with class V are the proliferative forms of LN. [7, 8] For the purpose of this investigation, we defined pure proliferative LN (PPLN) as pure class III or class IV only while MPLN comprises combinations of class III & V or class IV & V.

When compared to pure membranous LN, mixed membranous LN found to have worse long-term outcomes, specifically in terms of patient survival and progression of renal disease.[5, 9–12] However, few studies have compared outcomes in individuals with MPLN and PPLN, and even fewer studies have been to examine the comparative outcomes of MPLN and PPLN under the new ISN/RPS classification.[13]

The aim of this study was to compare the clinical presentation and short-term outcomes defined as ESRD and complete remission in individuals with biopsy proven MPLN vs. patients with PPLN. In addition, clinical and laboratory results from baseline were used to identify clinical predictors of outcomes in MPLN and PPLN. We performed prospective data analysis of the kidney biopsy database in a large tertiary healthcare system using retrospective data. Our hypothesis was that individuals with MPLN would have worse short-term outcomes than those with PPLN.

## Materials and Methods

### Study Population

We identified lupus nephritis patients from a native renal biopsy registry (n = 1204) at Emory University Hospitals that contained records of renal biopsies between the years 2000 and 2011. The biopsies in the native renal biopsies were identified from a central university database using the 2014 Current Procedural Terminologies (CPT) code 50200 (percutaneous renal biopsies), in the absence of a V42 (kidney transplant) International Classification of Disease (ICD-9) code recorded at the time of renal biopsy. Transplant patients were also excluded subsequently by reviewing the electronic medical records. Individuals were then separated into sub-classes of LN by reviewing of the renal biopsy report by two nephrologists. The Emory IRB approved this study (IRB#00065305). The IRB exempted the study from getting informed consent from participants and patient records/information was anonymized and de-identified prior to analysis.

### Measurements

We obtained patient age at biopsy, gender, and race/ethnicity as part of the biopsy registry from the central university database. We used laboratory data from the central database to collect baseline values of serum creatinine, urine protein and urine creatinine, serum albumin, serum hemoglobin A1c, erythrocyte sedimentation rate (ESR), hemoglobin, anti-double stranded deoxyribonucleic acid (anti-dsDNA) titer, anti-neutrophils antibody (ANA) titer, anti-basement membrane antibody, human immunodeficiency virus (HIV) assays, serum complement C3 and C4 assays.

The central university database was also used to obtain clinical data on systolic and diastolic blood pressure and medications for all LN patients. We identified all patients with an ICD-9 diagnosis of hypertension (using the following ICD-9 codes: 401.0, 401.1, 401.9, 405.01, 405.11, 405.19, 405.91, 405.99, 642.00, 642.01, 642.02, 642.03, 642.04, 642.10, 642.11, 642.12, 642.13, 642.14, 642.20, 642.21, 642.22, 642.23, 642.24, 642.70, 642.71, 642.72, 642.73, 642.74).[14] In addition to identifying patients who received treatment regimen for induction or maintenance therapy for LN through the Emory central data warehouse, we also performed a chart review of all LN patients to confirm treatments. We calculated estimated glomerular filtration (eGFR) rate using the Modified Diet in Renal Disease (MDRD) equation.[15] All data were obtained at time of biopsy (±90 days) and at 1 year post-biopsy (±90 days). Data for serum creatinine and random urine protein and creatinine were also obtained at each patient visit subsequently after baseline up until 2 years post biopsy (±90 days).

We categorized the diagnosis of proliferative LN into PPLN and MPLN following a review of histopathology findings for all patients based on the International Society of Nephrology/Renal Pathology Society (ISN/RPS) and World Health Organization (WHO) criteria for classifying LN.[4]

#### Outcome measurements

End stage renal disease (ESRD) was defined as the presence of an ICD-9 diagnosis code (585.6) for ESRD or the onset of chronic renal dialysis indicated by a CPT dialysis code of 90989, 90993, 90945, 90947, 90999 or an ICD-9 dialysis diagnosis code of V56.0, V56.8 and V45.11. Chronicity of dialysis was defined as at least 3 months of continuous dialysis since onset. We verified this through an independent chart review of all dialysis cases. Time to ESRD was determined from the first renal biopsy diagnosing LN to an ICD-9 or CPT code indicating dialysis or ESRD.

Response criteria for complete remission was modified from the American College of Rheumatology Response Criteria for Proliferative and Membranous Disease Renal Disease in Systemic Lupus Erythematosus Clinical Trials 2006[16] criteria and was defined as (1) improvement of at least 25% in eGFR if baseline abnormal or increase in eGFR to ≥ 90ml/min/1.73 m^2^ and (2) urine protein: creatinine ratio (UPCR) less than 0.5. If no data was found for patient at 1 year after biopsy the patient was excluded from the logistic regression and Cox proportional hazards analyses.

### Statistical Analysis

Descriptive statistical analysis was performed using frequencies and percentages for categorical variables, while the mean and standard deviation or median and interquartile range were reported for continuous variables. Bivariate analysis was performed using chi-square tests (categorical variables) while T-tests and Wilcoxon rank-sum tests were performed for normally and non-normally distributed continuous variables, respectively. Associations with outcomes were assessed using time-to event analyses was performed using Cox proportional hazards model. The proportional hazard (PH) assumption was checked using log-log survival curves and Schoenfeld residuals and the PH assumption was met. Cumulative survival curves were generated using Kaplan-Meier curves and difference between curves for the exposure variable compared using log-rank test. Independent covariates were identified from the literature and assessed for multicollinearity. We utilized a stepwise regression by adding individual covariates to the existing model in a stepwise manner using a significance level of p<0.05. In addition, we forced variables which were not significant into the model if they were considered a priori confounders (related to the exposure and the outcomes of interest). However, we excluded variables that had little variation and/or too few observed outcomes in subgroups defined by the variable.

Models of complete remission was adjusted for age, gender, hemoglobin, ESR, treatment, eGFR, serum albumin, dsDNA titer, and UPCR. Models of time to ESRD were adjusted for age, hemoglobin, ESR, eGFR, serum creatinine, dsDNA titer, UPCR. Race and hypertension were excluded from the final models of both complete remission and ESRD because of the lack of variability in these variables, resulting in too few outcomes among subgroups. Gender was also excluded from the final model for ESRD because of the same reason.

Missing data for covariates were accounted for by utilizing the multiple imputation (fully conditional specification) method for missing data analysis.[17] We assessed the final model for confounding and influential observations after imputation. Hazard ratios and confidence intervals were reported for crude and adjusted models. A sensitivity analysis comparing estimates from the complete case model and imputed model was also done to assess robustness of the model. All data was analyzed using SAS 9.4 Inc. (Cary, NC) statistical software and statistical significance was set at α = 0.05.

## Results and Discussion

### Results

The mean age of the entire population was 33.4 years (Table 1). The population was predominantly female; 18.3% of the PPLN group and 14.6% of the MPLN patients were male. Overall African Americans were 71.8% of the entire population, 58.3% of the pure proliferative group and 80.2% of the mixed proliferative group. Majority of the individuals (80.1%), had been treated with either angiotensin converting enzyme (ACE) inhibitors, steroids or other immunosuppression, although this percentage was higher in patients with MPLN (84.4%) than those with PPLN (73.3%). The mean overall hemoglobin level was 10.3mg/dl (s.d = 1.78). Mean serum albumin was lower in the MPLN patients (2.23) than in the PPLN patients. (2.46). However, there was no significant difference in the median protein/creatinine ratio in the two groups, 2.84g in the PPLN group and 3.05g in the MPLN group. The median eGFR was 63.9ml/min and 61.7ml/min for PPLN and MPLN groups respectively (Table 1).

**Table 1:** Patient Characteristics by Lupus Nephritis Category.

Characteristic	Overall	Pure LN 60(38.5)	Mixed LN 96(61.5)	P-value
**Demographic Characteristics**				
Age* mean (s.d)	33.41 (11.6)	34.57 (13.0)	32.69 (10.6)	0.33
**Gender** n (%)				0.53
Female	131(84.0)	49 (81.7)	82 (85.4)	
**Race** n (%)				0.01
Other	31 (19.9)	17 (28.3)	14(14.6)	
*African Americans*	112 (71.8)	35 (58.3)	77(80.2)	
*Missing*	13 (8.33)	8 (13.3)	5(5.2)	
**Clinical and Laboratory Characteristics**				
Hemoglobin level* mean (s.d)	10.33 (1.8)	10.59 (1.8)	10.16 (1.7)	0.15
ESR* mean (s.d)	70.31 (31.0)	71.80 (73.0)	69.51 (32.0)	0.77
eGFR* median (IQR)	62.50 (55.5)	63.90 (53.0)	61.65 (55.6)	0.002
Serum Albumin* mean (s.d)	2.32 (0.65)	2.46 (0.6)	2.23 (0.7)	0.03
Serum creatinine* median (IQR)	1.20 (0.92)	1.20 (0.8)	1.20 (1.1)	0.36
DsDNA titer* median (IQR)	270.50 (910.0)	292.00 (1276.0)	266.00 (569.0)	0.01
**Treatment** n (%)				0.093
No	31 (19.9)	16 (26.7)	15 (15.6)	
Yes	125 (80.1)	44 (73.3)	81 (84.4)	
Urine Protein Creatinine Ratio* median (IQR)	2.91 (3.9)	2.84 (3.9)	3.05 (3.7)	0.89

*Baseline characteristic. ESR = Erythrocyte Sedimentation Rate, eGFR = estimated glomerular filtration rate, IQR = interquartile range, ESRD = End Stage Renal Disease, DsDNA = Double stranded deoxyribonucleic acid.

**Fig 1:**
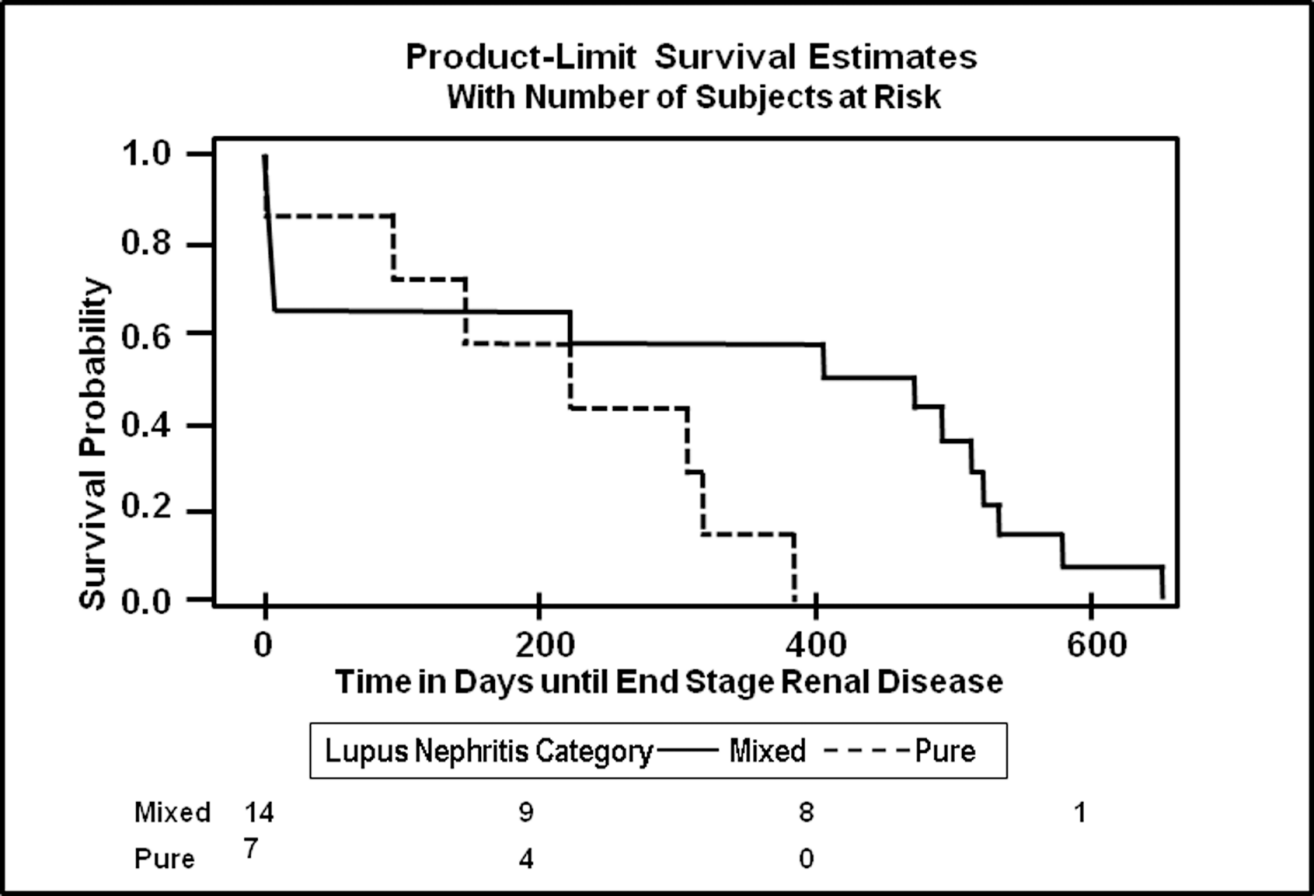
Kaplan Meier Plot of time to End Stage Renal Disease after Kidney Biopsy in Days by Proliferative Lupus Nephritis Category. Kaplan Meier plot showing the time to ESRD in days in individuals with MPLN and PPLN

Over a median follow up period of 1.02 years 12.8% of patients achieved full remission, 15.0% in the PPLN group and 10.4% in the MPLN group (Table 2). There was no association found between types of proliferative LN and remission, time to complete remission at one year did not differ significantly in the MPLN group when compared to the PPLN group in both the crude (HR = 0.82, 95% CI = 0.33, 2.02) and adjusted models (HR = 0.13, 95% CI = 0.01, 1.36). Fig 1 shows the Kaplan Meier curve showing time to remission for pure and mixed LN. When we used eGFR alone as a criterion for remission, we found that 36.4% (n = 32) individuals achieved remission and of these individuals, 34.4% (n = 11) had PPLN while 65.6% (n = 21) had MPLN (results not shown).

**Table 2:** Crude and Adjusted Hazard Ratios for Remission and ESRD in one year post biopsy diagnosis of Lupus Nephritis.

Characteristic	Complete Remission at 1year		ESRD^!^	
	Crude HR (95% CI)	Adjusted HR (95% CI)	Crude HR (95% CI)	Adjusted HR (95% CI)
LN Category				
*Pure*	Ref	Ref	Ref	Ref
*Mixed*	0.82 (0.33, 2.02)	0.13 (0.01, 1.36)	0.43(0.16, 1.14)	0.33 (0.09, 1.21)

! = End Stage Renal Disease. Complete remission adjusted for age, gender, hemoglobin, ESR, treatment, eGFR, serum albumin, DsDNA titer, UPCR. ESRD adjusted for age, hemoglobin, ESR, eGFR, serum creatinine, DsDNA titer, UPCR.

When we explored other factors that were associated with remission at two years in our entire population, only baseline eGFR was found to be associated with remission at 2 years. The crude hazard ratio for a 1-ml/min/1.73 m^2^ change in eGFR, was 0.96 (95%CI: 0.95–0.98) was associated with 4% lower risk of remission in crude models (HR = 0.96 95% CI = 0.95–0.98); in the adjusted model, each 1-ml/min/1.73 m^2^ increase in eGFR, was associated with 10% lower risk (HR = 0.90 95% CI = 0.84, 0.98) (Table 3). In the entire population of proliferative LN, female sex was found to be associated with complete remission in the crude model (HR = 0.37, 95% CI = 0.14, 0.97) but not in the adjusted models (HR = 0.43, 95% CI = 0.06–2.89).

**Table 3:** Crude and Adjusted Hazard Ratios of other Factors Associated with Remission and ESRD in one-year post biopsy diagnosis of Lupus Nephritis.

Characteristic	Complete Remission at 1year		Characteristic	Time to ESRD	
	Crude HR (95% CI)	Adjusted HR (95% CI)		Crude HR (95% CI)	Adjusted HR (95% CI)
**Age**	1.00 (0.96, 1.05)	0.94 (0.82, 1.08)	**Age**	1.04(1.00,1.07)	1.03 (0.98,1.08)
**Female**	**0.37 (0.14, 0.97)**	0.43 (0.06, 2.89)	**Female**^#^	1.57(0.45, 5.50)	
**Hemoglobin**	0.91 (0.67, 1.22)	2.04 (0.58, 7.14)	**Hemoglobin**	0.91 (0.63,1.32)	0.96 (0.56, 1.65)
**ESR**	1.01 (0.99, 1.02)	0.99 (0.94, 1.04)	**ESR**	1.03 (0.99,1.06)	1.01 (0.97, 1.06)
**eGFR**	**0.96 (0.95, 0.98)**	**0.90 (0.84, 0.98)**	**eGFR**	**0.99 (0.97,1.01)**	**0.99 (0.95, 1.03)**
**Serum Albumin**	**0.38 (0.18, 0.82)**	0.07 (0.00, 2.25)	**Serum Creatinine**	1.07 (0.94,1.21)	0.79 (0.42, 1.48)
**DSDNA titer**	1.00 (1.00, 1.00)	1.00 (1.00, 1.00)	**DSDNA titer**	1.00(1.00, 1.00)	1.00 (1.00, 1.00)
**Urine Protein Creatinine Ratio**	1.12 (0.92, 1.55)	0.88 (0.27, 2.81)	**Urine Protein Creatinine Ratio**	1.15 (1.00,1.32)	1.18 (0.94, 1.47)

^#^ Gender was not included in the final model for ESRD because almost all individuals with ESRD were female.

Over a median follow up of 1.52 years a total of 16.7% of individuals with PPLN developed ESRD, while 11.5% of those with MPLN developed ESRD (Table 2). When we examined the association between the type of proliferative nephritis and ESRD, using the PPLN as the reference group, there were no significant differences in the hazards of developing ESRD between the two groups (adjusted HR = 0.33, 95% CI = 0.09, 1.21) (Table 2).

**Fig 2:**
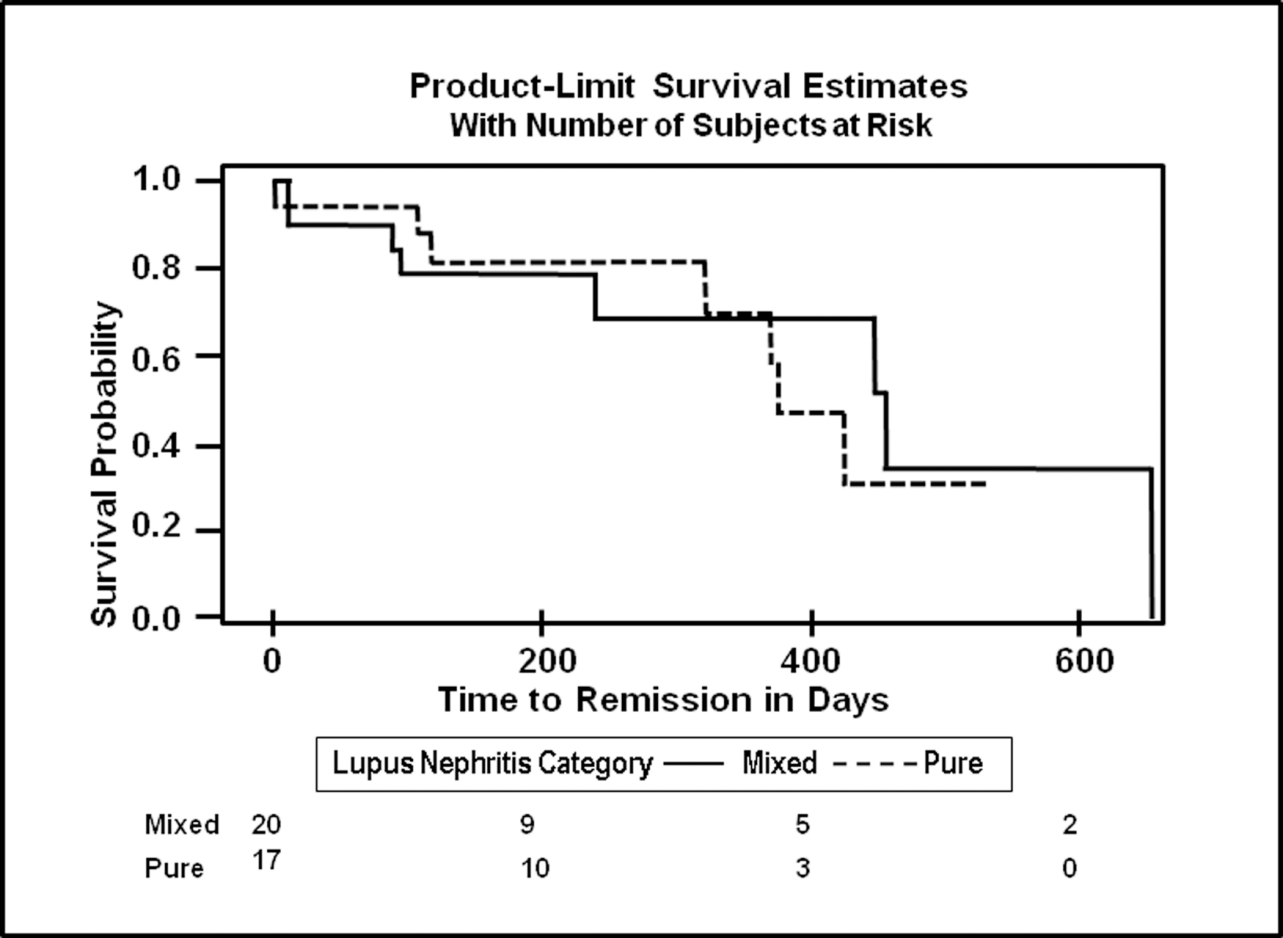
Kaplan Meier Plot of time to Complete Remission by Proliferative LN Category. Kaplan Meier plot showing the time to ESRD in days in individuals with MPLN and PPLN

Fig 1 shows the Kaplan Meier curve showing time to complete remission for MPLN vs PPLN. Fig 2 shows the time to ESRD for MPLN vs PPLN with was no significant difference in the MPLN vs PPLN groups.

## Discussion

In this study, 156 patients with proliferative LN, from an urban tertiary academic medical center in the southern US were compared by LN classification. No association between proliferative LN group and time to ESRD or complete remission was observed. This implies that there is no association between the histological category of proliferative LN and ESRD or complete remission respectively. However, baseline eGFR was significantly associated with remission among all patients.

The WHO, in 1982 initially divided LN into distinct patterns of injury and the degree of microvascular inflammations including the severity of the glomerular microvascular inflammation seen in LN I predictive of morbidity and mortality.[4], [7, 18–22] These groups include focal segmental glomerulonephritis (FSGN; category III), diffuse proliferative glomerulonephritis (DPGN; category IV), and complex membranous glomerulonephritis (categories Vc, Vd).^8^ In 2005, the new International Society of Nephrology and Renal Pathological Society classification introduced vital modifications in order to accommodate the quantitative and qualitative differences between class III and IV.[4] It also emphasized that the combinations of membranous and proliferative glomerulonephritis (i.e. class III and V or class IV and V) should be reported individually in the diagnostic line.[4]

The conventional wisdom has been that the presence of proliferation in the histopathology of LN is indicative of a worse prognosis, thereby warranting more aggressive treatment therapy in individuals with Lupus class III and class IV.[19, 20, 23–27] Our study findings are similar to that of Boneparth et al. done in children. Boneparth et al found no significant difference in complete remission defined as protein/creatinine ratio of < 0.5 and hematuria was defined as < 6 RBC/hpf on urinalysis in individuals with PPLN or proliferative LN or MPLN (Membranous + proliferative lupus nephritis); neither was there a difference in the subgroups at six months (class III or IV LN).[13]

Our results differ from other studies that have shown that the prognosis in individuals with MPLN is worse than that of PPLN.[11, 19] Sloan et al studied 85 patients with pure SLE utilizing the WHO 1982 classification. Najafi et al showed that there was a difference in remission rates over ten years for individuals with category IV LN compared to individuals in class Vc and Vd which we have categorized as MPLN (60% vs 27% respectively).[19] Najafi et al. examined remission rates of severe LN under an oral cyclophosphamide treatment regimen and found that only 27% of patients with class IV+V LN by ISN/RPS criteria entered remission after 120±65 months of follow-up, compared with 51% of patients with class IV LN.[19] The differences in results between our study and that of Najafi et al may be due to a variety of reasons. Firstly, the population was predominantly white (63%), compared to our study which was predominantly African Americans (71.8%).[28] Secondly, patients were treated with prednisone and cyclophosphamide with or without plasmapharesis in the study by Najafi et al, while our patients had a variety of treatment protocols including Cellcept. The follow-up period in the Najafi et al study was longer, with a mean follow up of 120 +/- 65 months compared to our study that had a median follow up of 1.02 years for remission and 1.52 years for ESRD.[28]

Sloan et al studied differences in outcomes using the 1982 WHO classification and found that the pure forms of class V LN (class Va or Vb) had a better outcome than proliferative class WHO Vc or WHO Vd. The 10-yr survival rates were 72, 48, and 20% respectively with significant differences between Va and Vb and Vc > 50% and Vd despite no differences in baseline characteristics in the three groups.[11]^,^ [29]

Our cohort was predominantly female and African American with a mean age of 33.4 similar to other studies with patients with LN.[19] We also found that baseline eGFR was associated with decreased time to remission as has been seen in previous studies. [30]

Our study was inherently limited by its retrospective nature and a relatively small sample. In addition, the different definitions of complete remission may have led to misclassification and our inability to detect a significant difference between the two groups. By combining the two proliferative groups of LN together, we may have obscured vital morphological differences that correlate with clinical differences and prognostic determination. In addition, we did not record outcomes such as mortality, so these cases were not censored if they occurred. Other individuals may also have been lost to follow-up and this may not have been captured. The codes used were specific for Emory data. If dialysis was received outside of Emory, it would not be in the system. Even after a thorough chart review, some of this information may be inaccurate. Also the presence of residual confounding due to unmeasured or unknown confounders is a limitation. Our study is also limited by the short-follow up period for our outcomes remission and ESRD. Our median follow–up was 1.02 years for remission and 1.52 years for ESRD, compared to other studies that had a median follow up of 120 months.[28]

## Conclusion

This study warrants follow-up investigations to elicit the similarities and differences between the classes of proliferative LN and this will ultimately inform treatment strategies that will be unique to addressing the underlying mechanistic disease process.
